# Spatio-Temporal Analysis of Natural and Anthropogenic Arsenic Sources in Groundwater Flow Systems

**DOI:** 10.3390/ijerph15112374

**Published:** 2018-10-26

**Authors:** Claudia Avila-Sandoval, Hugo Júnez-Ferreira, Julián González-Trinidad, Carlos Bautista-Capetillo, Anuard Pacheco-Guerrero, Edith Olmos-Trujillo

**Affiliations:** Doctorado en Ciencias de la Ingeniería, Universidad Autónoma de Zacatecas, Campus UAZ Siglo XXI, Carretera Zacatecas-Guadalajara Km. 6, Ejido la Escondida, C.P. 98160 Zacatecas, Mexico; claudia_ivetthe@hotmail.com (C.A.-S.); hejunez@uaz.edu.mx (H.J.-F.); baucap@uaz.edu.mx (C.B.-C.); anuard.pacheco@uaz.edu.mx (A.P.-G.); editholmostru@gmail.com (E.O.-T.)

**Keywords:** arsenic, natural background levels, indicator kriging, flow systems, groundwater

## Abstract

The presence of arsenic in groundwater constitutes a hazard for the environment and human health, and the determination of its source has become a global challenge, which can be approached by defining the natural background levels (NBL) in conjunction with the indicator kriging method, with the aim of delineating anthropogenically contaminated areas. However, having a unique value of NBL for large areas can generate interpretation errors. This research integrates the determination of the flow systems present in the Calera Aquifer, and the definition of the natural background levels in each flow system by making estimation maps in ArcGIS using two databases, 10 years apart, to evaluate the spatio-temporal variation of arsenic in groundwater. The results indicate a notable increase in the probability of exceeding the arsenic NBL, mainly in the intermediate flow, which may be due to movement resulting from mining activities as well as a mixture of regional and intermediate flows caused by the extraction of water for agriculture and drinking water supplies. The presented values exceed the maximum limits allowed for human consumption, as stated by the World Health Organization.

## 1. Introduction

The natural and anthropogenic occurrence of arsenic in groundwater has become a concern in human health studies [1]. It is estimated that millions of people are at risk of the adverse effects of arsenic exposure through drinking water [2], which results in chronic poisoning that leads to changes in skin pigmentation and thickening, as well as various types of cancers of the skin, lungs, bladder, and kidney. Thus, in 1980, the international agency for cancer research classified arsenic in group one, as a carcinogen for humans beings [3].

Arsenic in groundwater has been identified in 105 countries in the world, and the population exposed to a concentration greater than the reference value indicated by the World Health Organization (WHO) is estimated to be to >200 million worldwide [4]. The natural occurrence of high values of arsenic in groundwater has been reported in different geologically young aquifers around the world [5,6,7]. Some research reports that the pollution of groundwater by arsenic may be due to agricultural activities in connection with the application of fertilizers and pesticides [8,9], as well as by mining activities [6,10,11].

Variation in the concentrations of arsenic in groundwater may arise in response to natural or anthropogenic factors. Natural variation in arsenic concentrations might be expected to occur in response to climatic and seasonal changes, such as wet and dry periods, although these factors have rarely been directly linked to variation in arsenic concentrations. Anthropogenic factors that may affect arsenic variability include land development, the addition of solutes to groundwater systems, or human-induced flow-system changes, including well development, groundwater pumping, and aquifer storage and recovery [1]. It is reported that the concentration of Arsenic (As) in groundwater may vary laterally, from unsafe to safe, within a range of 10–100 m, which often constrains the identification of safe regions and exact As mobilization mechanisms, and the spatial and temporal variation in groundwater As concentrations can have severe consequences with respect to the potential As exposure of people drinking presumably safe water [5]. In the treatment of environmental data, multivariate techniques have been used for a long time in the assessment of the degradation and/or spatial and temporal contamination of groundwater caused by natural or anthropogenic factors. Cluster analysis (CA) and principal component analysis (PCA) are important tools for the multivariate techniques, and they are applied to determine the dominant interrelationships among the variables to understand the processes responsible for controlling the chemistry of groundwater [12]. Multivariate statistics have been used extensively to reduce the complexity of large-scale data sets [13], as well as to classify and interpret the hydrogeochemical processes that occur in groundwater [6]. Several works have demonstrated the utility of a combination of principal component analysis and hierarchical cluster analysis in the determination of flow systems [14].

The assessment of the qualitative status of groundwater bodies is related to the definition of natural background levels (NBL) and threshold values (TV). The former are mainly related to the hydrogeochemical environments of the aquifer, while the latter are associated with the public health problem. Having a single NBL value for an element within a study area does not allow for the representation of the local variation in the geochemical and environmental conditions. In fact, it could generate a considerable uncertainty in the definition of the natural presence of a contaminant in an anthropogenic source [15]. The implementation of the NBL concept for groundwater quality management would benefit from the use of an integrated methodology that would allow for a clear identification of the distribution of the concentrations of a substance within a body of groundwater. The indicator kriging (IK) method, a non-parametric interpolation method based on transformed data for producing a set of binary value data, allows us to estimate the probability that the concentration of an element in a given place exceeds a predefined threshold value [16].

The main aim of this study is to propose a methodology that integrates the identification of flow systems and their relationship with natural background levels, allowing the spatio-temporal behavior of arsenic to be understood. It has the following specific objectives: (1) To define the flow systems in the historical series, using the main components; (2) to determine the natural background levels in each defined flow system to identify the anthropogenic or natural origin of arsenic; and (3) to evaluate the variation of arsenic’s temporal space, considering the natural background levels and using the indicator kriging method. The novelty of this work is its methodology, which integrates flow systems and the definition of natural background levels. It is considered that, by incorporating the temporal component, it will be possible to explain the evolution of arsenic in groundwater, with greater certainty in the definition of the sources of natural degradation and anthropogenic contamination. This will allow decision makers to assess the current status of the aquifer and determine if its use is feasible. The methodology will be applied to the case of the Calera aquifer, in the state of Zacatecas, Mexico, due to its antecedents of high levels of arsenic [17].

## 2. Materials and Methods

### 2.1. Study Zone

The Calera aquifer is situated in the central zone of the state of Zacatecas, and has an area of 2087.6 km^2^, with an average rainfall of 450 mm/year, which is due to its rainy season occurring between June and September, and a potential evapotranspiration of 1900 mm/year. This aquifer is the main source of fresh water for agricultural and industrial activities, with an average annual extraction of 125 mm^3^ [18]. It is located in the volcanic terrain of the Sierra Madre Occidental, in the south of a regional graben structure that gives rise to the Calera endorheic basin, characterized by ephemeral streams that are dry for most of the year. The Zacatecas Formation (maximum elevation 2700 m.a.s.l.) and the Chilitos Formation are linked to a flat area (2010 m.a.s.l.) in the south-central part and another flat area (2100 m.a.s.l.) in the north, and their floors are between 10 cm and 50 cm in depth. In general, they are considered arid, and the average annual temperature is between 18 and 20 °C, with a precipitation that varies from 400–450 mm. There is an important surface run-off, with small intermittent currents, which is towards the center of the study area and continues towards the north, with some water surface reservoirs of a reduced capacity, but of great importance for the area. This zone covers two important cities in the state of Zacatecas, where the main economic and social activities are established [17].

The recharge of the aquifer comes mainly from the pluvial precipitation, which occurs over the mountain ranges and hills of the basin, and from transmission losses from the flash floods in the ephemeral streams. In smaller proportions, the water precipitated in the valley recharges vertically in relation to the aquifer. Finally, another volume comes from the returns of irrigation by pumping. The artificial discharge is conducted by pumping wells; naturally, a small volume is drained by the underground flow to the Santa Ana and Sedano lagoons. The preferential direction of the underground flow is from south to north, with an entry in the east and west portions, within the limits of the mountains and hills [19]. The animal husbandry is a complementary occupation in the metropolitan area of Zacatecas-Guadalupe. On the other hand, mining activities have gained strength and become a major influence on the quality of groundwater over the past 30 years. Due to the industrial activity present in the area, a large amount of groundwater is extracted, and 50% of the total potable water, supplied to Calera, Morelos, and the metropolitan area of Zacatecas-Guadalupe, comes from the central and western part of the Calera aquifer [17].

#### Geology

**Figure 1:**
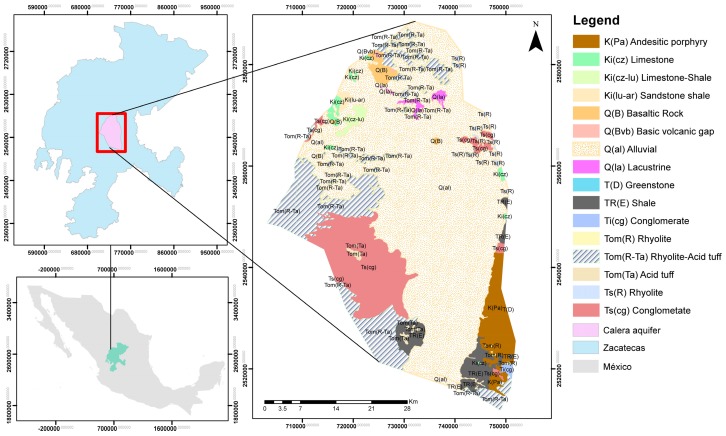
Geology of the study area. Geographic coordinates system in WGS84.

In the Calera aquifer, the alluvium extends between tectonic pillars, which consist mainly of metamorphic rocks from the Zacatecas and Chilitos formations (Figure 1). To the west are the massive sulphide deposits, which evolve towards the Las Pilas Complex, that Francisco I. Madero developed into an arch of islands with intense mining activities. Geophysical surveys and direct drilling show that the aquifer is only semi-confined locally by interspersed clay layers [17].

The lithology encompasses pyroclastic volcanic rocks, subvolcanic rocks, basaltic-andesitic, and metasedimentary terrigenous lavas. The distribution of volcanic rocks, located in the western zone from south to north, is the most extensive in the aquifer, and it includes an irregular block of approximately 62 km^2^, located to the northwest of the town, Francisco I. Madero. The sub-volcanic rocks are restricted to small extrusions located in the vicinity of the City of Zacatecas in the Cerro de la Bufa to the south of Cerro del Padre and to the north in the Cerros La Sierpe, Magistral, Calavera, and Calicanto [20].

### 2.2. Databases

The evolution of groundwater quality was analyzed for a period of 10 years through two databases for different periods of time and in different wells; both databases are presented in the Supplementary File (Table S1). The first was in 2005, with a total of 99 samples [21], and the second was conducted in 2015, with 173 samples [17], of which 35 are used for human consumption. The depth of the monitored wells was measured with a water level probe, which varied from 5 to 300 m; the average water depth was 85 m, and the electrical conductivity, pH, temperature, dissolved oxygen, and alkalinity were sampled in situ. In 2005, it was also analyzed, Redox potential (Eh), by means of a combination electrode, P100C-BNC, with values ranging from −67 to 415 mV. In the case of As, major ions, and tracer elements, the samples were filtered (0.45 μm membrane filters) and acidified (1% *v*/*v* HCO_3−_) in the field. The F^−^ samples were not treated. The analytical determinations were carried out in the Environmental Engineering Laboratory of the Autonomous University of Zacatecas and the Laboratory of the Autonomous University of San Luis Potosí, and the As was analyzed by atomic absorption spectrophotometry (Thermo Scientific ICE AA 3300, Waltham, MA, USA), with hydride generation as well as the major ions, Ca^2+^, Na^+^, K^+^, and Mg^2+^. Calibration and validation for atomic absorption spectrophotometry were performed using a certificated standard. The ion balance ranged between ±7%, the parameters were determined under the guidelines described in the Standard Methods for the Examination of Water and Wastewater (APHA-SMWW 2006).

### 2.3. Determination of Flow Systems

The flow of groundwater is essentially controlled by geological and hydrogeological factors, which are represented in terms of the horizontal distance of movement and depth [22]. These hydraulic characteristics, and the idea that, in an aquifer, there are zones with a specific water quality called hydrochemical facies [23], form the basis for using a methodology to differentiate them. It is therefore feasible to establish a conceptual model for a multivariate analysis of: (a) The variables that control the regional and intermediate flows; and (b) the differentiation of flow systems through the analysis of the principal components (PCA) and cluster analysis [14].

The determination of the data flow systems was carried out through a multivariate analysis, using IBM SPSS Statistics, Version 20.0 (IBM Corp., Armonk, NY, USA) to determine: (a) The analysis of the principal components (PCA); and (b) the cluster analysis based on the results of the PCA. The PCA is a multivariate statistical procedure designed to classify correlated variables (in this case, dissolve chemical elements in groundwater) and reduce them to a few factors that can be interpreted more easily [24]. With the analysis of the main components, different associations between variables and samples were determined. When obtaining the cluster number, a k-means grouping was used to classify the samples with greater distinction.

### 2.4. NBL Estimation

Nowadays, the assessment of the qualitative status of a groundwater body is intrinsically related to the definition of natural background levels (NBLs) and threshold values (TVs), which are usually groundwater quality standards, defined according to the groundwater use [16]. The natural background levels (NBL) are defined as the level of concentration in water, controlled by natural geological, biological, and atmospheric processes and not influenced by human activities [18]. The selection method consists in selecting only those samples that are not influenced by anthropogenic activities, such as mining and agriculture, since natural groundwater concentrations are characterized by a concentration range, which is defined by certain percentiles of the natural component distribution [25]. The sample sets and the residual data set are eliminated, and the value is chosen, which is generally represented as the 90th, 95th, or 97th percentile. The value is chosen according to the degree of knowledge of the conceptual model and the hydrogeochemical system. All concentrations exceeding this level must be attributed to anthropogenic sources [16]. In this case, the 90th percentile was used because there are less than 60 samples in the data set [26] presented in each flow system.

The evolution of arsenic in groundwater can be evaluated by determining the natural background levels, in which samples that may be contaminated by anthropogenic processes are discarded by a preselection, as proposed by Hinsby et al. [27], using the nitrate modifications proposed by Preziosi et al. [28], with the following criteria:

(1) Chloride concentration as a salinity indicator: Samples with [chloride] N > 200 mg/L were discarded;
(2) concentration of nitrates and organic pollutants, as an indicator of the human impact in oxidized groundwater (DO > 2 mg/L o Eh < 100 mV): Samples with [NO_3_] > 50 mg/L, or with total organic contaminants > 0.05 μg/L, were discarded;
(3) oxidation capacity (OXC) [29], as an indicator of the human impact in the reduction of groundwater (DO < 2 mg/L, Eh < 100 mV o Fe (II) > 0.2 mg/L, [22]. OXC (meq/L) was calculated as 7 [SO4] + 5 [NO_3_], with the concentration of the species in [mmol/L], and samples with OXC > 2 meq/L were discarded; and
(4) ammonium: Samples discarded with NH4 > 0.5 mg/L under reducing conditions.

The NBL was established as the 90th percentile of the samples that are not contaminated.

### 2.5. Method of Indicator Kriging

The link between arsenic and complex hydrogeochemical processes is not yet understood, and geo-statistics, such as the measurement of inverse distances and natural neighbors through the analysis of variograms, is a useful tool for addressing this problem [15].

The conventional variograms and the geostatistical analysis are limited by the properties of the algorithms, the main problems being (i) normality (although the techniques are quite robust) and (ii) the independence of the standard value of ordinary kriging (OK) in the data values. The tool that allows these limitations to be overcome is the indicator kriging [16], which allows for the designation of several thresholds that allow the data to be transformed into a set of binary variables.

The calculation of the variogram is made according to a set of pairs of points, separated by an increased distance, h, by means of the following expression:

(1) $$\gamma \left(h\right)=\frac{1}{2n\left(h\right)}\cdot \overset{n\left(h\right)}{\underset{i=1}{\sum }}{\left[Z\left(X_{i+h}\right)-Z\left(X_{i}\right)\right]}^{2}\;i=1,\ldots ,n$$

where $Z\left(X_{i}\right)$ and $Z\left(X_{i+h}\right)$ are the values of the variable, *Z*, measured at the points, $X_{i}$ y $X_{i+h}$, respectively, and n(h). denotes the number of pairs of points, separated by a lag, h.

The most widely used theoretical model for adjusting the functions of the experimental variogram is the spherical model, which is expressed by the following equations:

(2) $$\begin{array}{c} \gamma \left(h\right)=∁[\frac{3}{2}\frac{h}{a}-\frac{1}{2}{\left(\frac{h}{a}\right)}^{3}]\;if\;h<a\\ \gamma \left(h\right)=∁\;if\;h\geq a \end{array}$$

where ∁ is the threshold (in most cases, it is equal to the variance of the sample) and a is the range, which is a distance beyond which the variable is not correlated. Another frequently used model is the Gaussian, which, in contrast to the spherical model, only asymptotically reaches the threshold value for which it does not show a defined range. It assumes a parabolic behavior near the origin and is characteristic of the data that show a high degree of spatial continuity. The formula of the Gaussian variogram is:

(3) $$\gamma \left(h\right)=∁\left[1-exp\left(-\frac{3h^{2}}{a^{2}}\right)\right]$$

However, one can define an effective range $\left(a^{\prime }=\;3a\right)$, which corresponds to 95% of the threshold $\left(y\;\left(a^{\prime }\right)\;=\;0.95C\right)$.

The transformation of the indicator, *I* (*X*), at the location. *X*, for the data of value, z(x), estimated by the limits, k, of cut, *z*k, is defined by the following expression [30]:

(4) $$I_{Z_{c}}\left(x\right)=\{\begin{array}{c} 1\;if\;Z\left(x\right)\geq Z_{c}\\ 0\;otherwise \end{array}$$

The results of the indicator kriging represent the possibility that the threshold value is exceeded, and these can be values between 0 and 1 [16].

## 3. Results and Discussion

### 3.1. Flow Systems

Lithology and climate generally determine the type of groundwater. Even if the lithology is similar, the degree of water-rock interaction will be determined by distance and travel time [6], however, diverse geochemical processes and the mixture modify its hydrochemical composition. An exploratory statistical analysis can provide valuable information and assemble samples with common features, which can yield statistical relationships that are expected to predict the hydrogeological processes that occur in the system [13]. To identify the present flows, a statistical analysis was performed for each set of data for the years, 2005 and 2015.

A matrix of correlations was generated to represent the statistical relationship between two or more variables by calculating the Pearson correlation coefficient in the year, 2005 (Table 1). The variables, redox potential (EC) and total dissolved solids (TDS), are closely related to the dependence on ionic solutes, and with Na^+^, a major constituent present in the groundwater TDS. In addition, a close relationship was found with HCO_3−_ due to the high solubility of limestone or dolomite carbonate minerals. The hardness is closely related to Ca^2+^ and Mg^2+^ cations [31]. The results for the year, 2015, are shown in Table 2. In general, similar results to those of 2005 were found; there is a relation between TDS, Na^+^, Mg^2+^, and Cl^−^.

#### Principal Component Analysis

The PCA results are shown in Table 3 for the years, 2005 and 2015. For the year, 2005, the parameters identified in C1 were CE, TDS, HCO_3_^−^, and Na^+^, and those identified in C2 were the hardness, Ca^2+^ and Mg^2+^. In C3, the temperature, pH, and Eh were identified, as well as in C4 K^+^. In 2015, C1 shows a positive relationship between TDS, Ca^2+^, Mg^2+^, and Cl^−^, and in C2, only the Na^+^ was identified. In C3, Li and HCO_3_^−^ were identified.

With the results of the PCA, a cluster analysis was performed to group the samples into groups that allow the flow systems for the years, 2005 and 2015, to be determined, and the results are shown in Table 4 and Table 5, respectively.

The average content of arsenic and fluorine is higher in all flows for 2015, representing a greater risk of the contamination of these ions due to the movement in the flows, which is reflected in the increase in temperature in all flow systems for this year.

In the year, 2015, all the values of F- were higher than in 2005, which may be due to an intense agricultural activity, where the use of phosphate is a common practice. Likewise, water with sodium characteristics causes high values of F, which is reflected in the local flow in the year, 2015.

**Figure 2:**
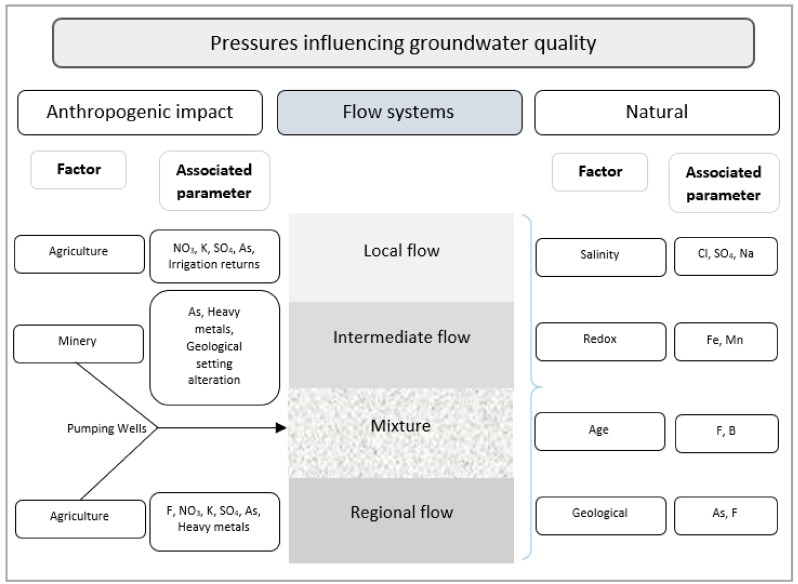
Conceptual model of the pressures influencing the groundwater quality of flow systems. Modified figure of [27].

A conceptual model of pressures influencing the groundwater quality of flow systems is shown in Figure 2. It can be seen that the local flow is influenced by the agriculture because there is return irrigation causing high values of As; also, parameters, like NO_3_, K, and SO_4_, can have a negative impact on the quality of groundwater. The intermediate flow can be affected by mining activities, since the mine’s own processes can alter the geological environment, causing an appropriate environment for the movement of arsenic. Agriculture can affect the quality of groundwater by using fertilizers and pesticides, elements, such as F-, and phosphate, which can alter the quality of water. Due to the pumping of water for potable water use and agricultural and mining activities, intermediate and regional flows are mixed, causing high concentrations of arsenic, which constitutes a risk for the water supply of the population. The groundwater is influenced by natural aspects, such as salinity (Cl, SO_4_, Na), redox conditions (Fe, Mn), age (F, B), and geology (As, F).

**Figure 3:**
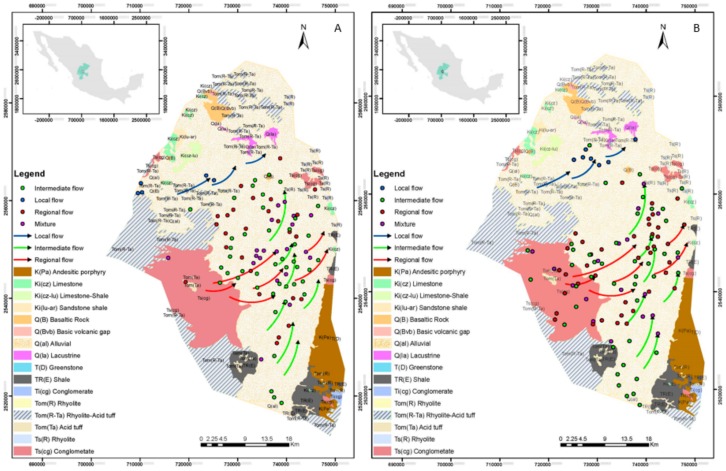
Geology and corresponding wells in each flow system in the years, (**A**) 2005 and (**B**) 2015.

Figure 3 shows the corresponding wells for each flow system. The lines correspond to the direction of each of the flows determined by a multivariate analysis; the results of the determination of the flow systems in this investigation show a direction of flow different from that shown by Navarro et al. [17]. The system flow theory used in the current study is described by Tóth [32], which considered the groundwater flow distances and the geochemistry of water.

**Figure 4:**
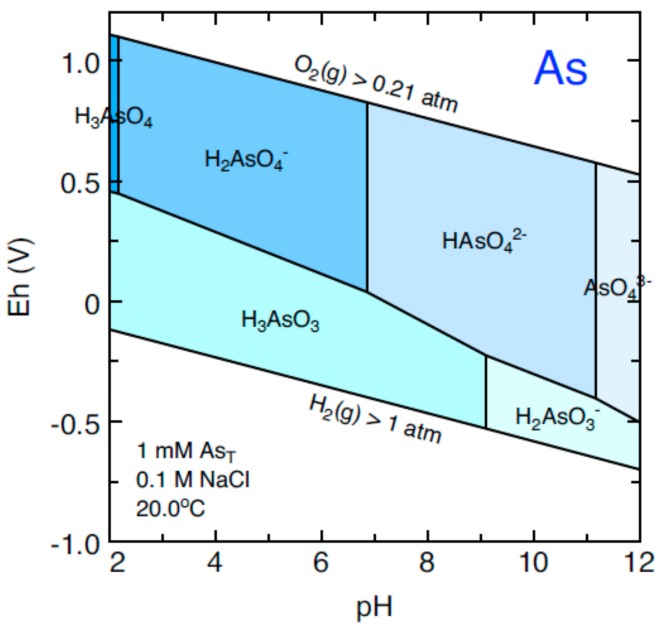
Redox potential(Eh)−pH diagram of aqueous As species in a system containing As [33].

Arsenic occurs in the environment in several oxidation states. In the water, it is mostly found as inorganic forms, arsenite (+3) and arsenate (+5); under oxid conditions at thermodynamic equilibrium, aqueous As is dominated by arsenate oxynions (H_2_AsO_4_^−^ or HAsO_4_^2−^ depending on pH conditions). Under reducing conditions and over a wide range of pH values, the uncharged arsenite species, H_3_AsO_3_, predominates (Figure 4) [33]. The 2005 data show Eh and allow the determination of the species of arsenic present in the Calera aquifer. The corresponding species for all flow systems is H_2_AsO_4_^−^. This means that under oxidizing conditions the arsenic present in the silicates [34] is being oxidized mainly due to the mining activity in the southern part for the intermediate flow and mixing. Finally, in the alluvial parte, there are oxide-reduction processes.

### 3.2. Natural Background Levels

The natural arsenic background levels for 2005 and 2015 were set as the 90th percentile of the non-contaminated samples, and the values are shown in Table 6. The reference value that is taken is the maximum arsenic limit allowed by WHO, which has a value of 10 µg/L.

The NBLs show a higher value than the reference values for almost all of the flows for the two years, except for the local flow in the year, 2005, which has a value of 4.92 μg/L. This means that, whatever the origin of the arsenic, exceeding the reference value represents a risk for the population, and in the same way, it is important to note that, in the year, 2015, higher values were obtained in all flow systems.

In 2005, the greatest value was presented in the regional flow, unlike in 2015, in which a higher value was obtained in the intermediate flow. With the values of the reference levels, an interpolation was made of the distribution of As, fitted by a spherical model, as shown in Table 7.

### 3.3. Estimation Maps of As

**Figure 5:**
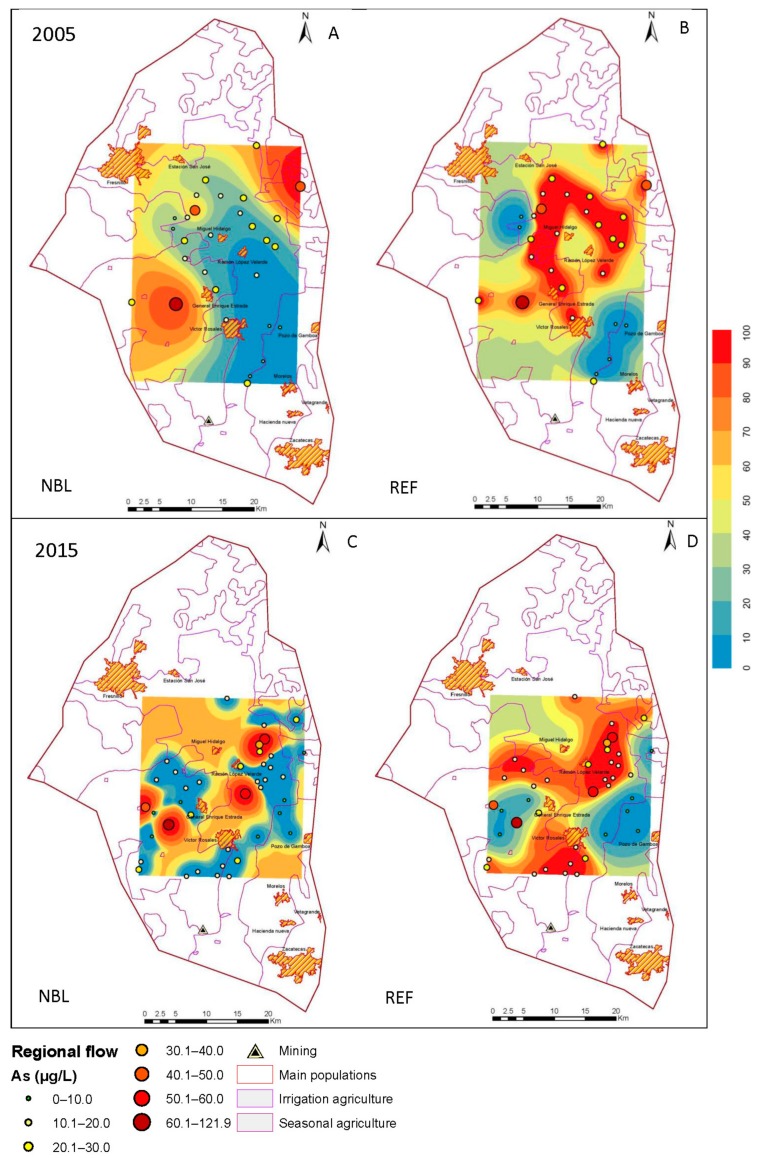
Maps of probability of exceeding the natural background level (NBL) and the reference values (REF) for 2005 (**A**,**B**) and 2015 (**C**,**D**) in the regional flow.

**Figure 6:**
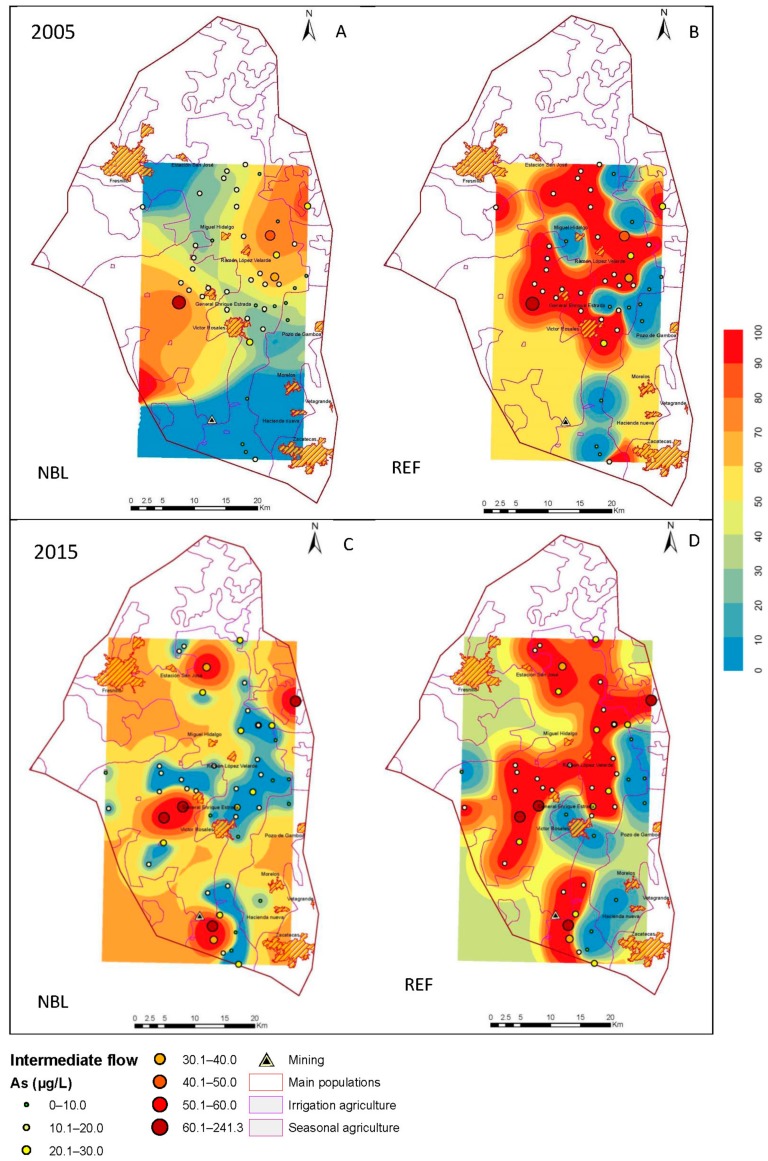
Maps of probability of exceeding the natural background level (NBL) and the reference values (REF) for 2005 (**A**,**B**) and 2015 (**C**,**D**) in the intermediate flow.

The behavior of the NBLs, in combination with the indicator kriging, allowed maps of estimations to be developed in the Geographic Information System, ArcGIS 10.0 (Environmental Systems Research Institute Inc.: RedLands, CA, USA), and the results are shown in Figure 4, Figure 5 and Figure 6. In each section, the corresponding estimation maps are shown in the years, 2005 and 2015, showing the prediction maps for the natural background levels (NBL) and the reference values (REF). The blue areas correspond to a 10% probability of exceeding the NBL and REF, and the red areas represent 100%.

The reference maps distinguish the areas where the TVs (determined by the permissible limits of water for human consumption) are violated due to natural processes of a geological origin from those that result from contamination.

#### 3.3.1. Regional flow

Since the distribution of volcanic rocks is the most extensive in the aquifer, they are located in the western zone, from south to north, and contain an irregular block of approximately 62 km^2^ located to the northwest of the town of Francisco I. Madero [20], where arsenic is found naturally in the aquifer Calera.

In the regional flow of 2005, a high probability of exceeding the natural background levels in the northwestern and southwest parts is observed (Figure 5), and in 2015, the probability is increased in the central part. In 2015, the probability of exceeding the reference values increased in the southern part, unlike in 2005.

#### 3.3.2. Intermediate Flow

The groundwater flows carry dissolved arsenic, as well as DOC, oxygen, sulfate, and competitive absorbents, and these factors influence the concentration of arsenic [35]. In this case, in the aquifer, there is an intermediate flow in the southern part, with an upward direction in the alluvial quaternary basin, and a notable increase is observed for the year, 2015. This may be related to the presence of mining activities, which influence this flow, thus facilitating arsenic mobility. The reference values surpass a large part of this flow (Figure 6).

#### 3.3.3. Mixture

**Figure 7:**
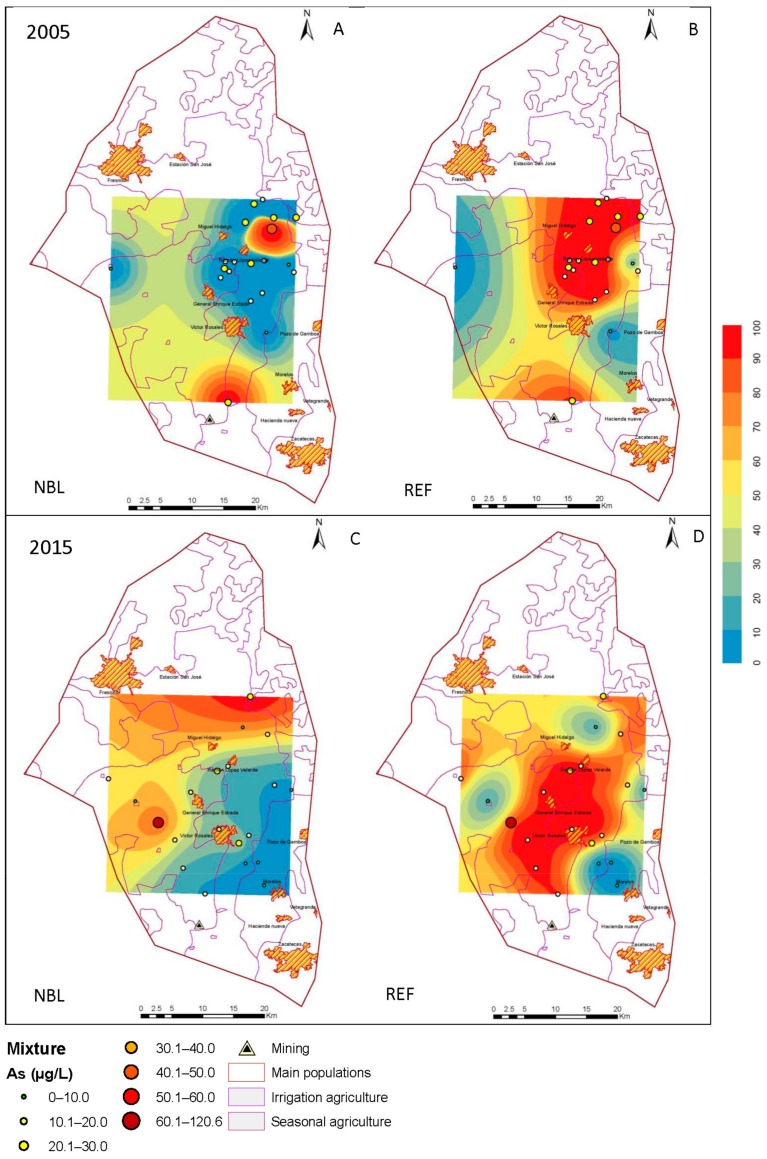
Maps of probability of exceeding the natural background level (NBL) and the reference values (REF) for 2005 (**A**,**B**) and 2015 (**C**,**D**) in the mixture.

The extraction of the groundwater in wells for agricultural activity and for the supply of drinking water generates a mixture of water from the regional and intermediate flows. In 2005, the probability of exceeding the NBL is concentrated in the northwestern part of the aquifer, and the reference values similarly cover a larger area. For 2015, a greater participation of the intermediate flow is observed, causing the reference values to be exceeded in the southern and northern parts (Figure 7).

For the purposes of this investigation, estimation maps of the local flow in both years were omitted due to the small amount of data presented, which did not show relevant results.

Arsenic is a natural constituent of the Earth’s crust, and ranks 20th in abundance in relation to the other elements. Arsenic can be presented in terrestrial and aquatic environments via natural geologic processes and anthropogenic activities. Atmospheric arsenic arising from coal burning has been cited as a major cause of lung cancer in parts of China and India. Values between 17 to 291 μg/day have been reported in food in different countries. Seafood accounts for 60–96% of the total dietary intake of arsenic, mostly in the form of arsenobetaine and arsenosugars, relatively non-toxic forms of arsenic. Other food sources are vegetables, mushrooms, grains, milk, chicken, and beef, which account for inorganic arsenic consumption. In non-arsenic endemic regions, the principal sources of inorganic arsenic in the diet are rice and chicken, which results in the accumulation (55–97 ng/g) of methylated arsenic compounds [36]. Drinking water has been reported to be the main route of arsenic exposure around the globe, and in some places of Zacatecas, it was estimated that 80% of the inhabitants could be exposed to arsenic levels higher than those recommended by EPA and the WHO [37].

## 4. Conclusions

A spatio-temporal analysis of the behavior of arsenic in the Calera aquifer was carried out through the interpretation of two databases of samples, taken 10 years apart, determining the flow systems for each period, and in each system, the natural levels were defined. In the background with the 90th percentile, prediction maps were finally made with the results, using the indicator kriging method in ArcGis to delineate contaminated areas.

Groundwater is not stable and concentrations of As change spatially and temporally. The identification of the flow systems allowed the contamination of the arsenic produced by mining activities in the intermediate flow for 2015, as well as the mixture of the regional and intermediate flows, caused by the extraction of water from wells that also have high arsenic values, to be identified.

In the Calera aquifer, most wells have values that exceed the limits allowed by the WHO, which makes it unfit for human consumption. This will cause serious human health effects given the fact that the consumption of drinking water is only one of the major exposure routes. Future research should be aimed at determining the impact of arsenic on the population and the factors that can facilitate the movement of arsenic in the aquifer. Since the wells destined for drinking water belong to the intermediate and mixed flows, a monitoring network must be carried out to control the extraction of water and thus supply the population in a safe way.

## Figures and Tables

**Table 1:** Correlation coefficients for the year, 2005. (T= Temperature, Eh = Oxidation potential, EC= Electrical conductivity, TDS = Total dissolved solids, and Har = Hardness).

Correlation	T	pH	Eh	EC	TDS	Har	HCO_3_^−^	Na^+^	K^+^	Ca^2+^	Mg^2+^
T	1.000										
pH	0.121	1.000									
Eh	0.338	0.370	1.000								
CE	−0.023	0.078	−0.119	1.000							
TDS	−0.148	0.089	−0.070	0.736	1.000						
Har	−0.312	0.147	0.163	0.135	0.631	1.000					
HCO_3_^−^	0.159	−0.050	−0.043	0.701	0.651	0.129	1.000				
Na^+^	0.158	0.041	−0.170	0.763	0.792	0.131	0.845	1.000			
K^+^	−0.151	0.159	0.144	0.325	0.496	0.263	0.289	0.423	1.000		
Ca^2+^	−0.244	0.117	0.215	0.010	0.516	0.901	0.055	0.065	0.370	1.000	
Mg^2+^	−0.311	0.144	0.051	0.251	0.651	0.877	0.182	0.184	0.083	0.628	1.000

**Table 2:** Correlation coefficients for the year, 2015.

Correlation	T	pH	TDS	Na^+^	K^+^	Ca^2+^	Mg^2+^	Li	HCO_3_^−^	Cl^−^
T	1.000									
pH	0.075	1.000								
TDS	−0.252	−0.346	1.000							
Na^+^	0.111	−0.219	0.721	1.000						
K^+^	−0.238	−0.308	0.560	0.573	1.000					
Ca^2+^	−0.290	−0.415	0.882	0.523	0.509	1.000				
Mg^2+^	−0.385	−0.268	0.808	0.337	0.311	0.791	1.000			
Li	0.231	−0.180	0.322	0.574	0.223	0.248	0.035	1.000		
HCO_3_^−^	0.006	−0.143	0.263	0.516	0.368	0.239	0.109	0.642	1.000	
Cl^−^	−0.313	−0.385	0.897	0.582	0.551	0.943	0.810	0.243	0.215	1.000

**Table 3:** Matrix of components, rotated for the years, 2005 and 2015.

2005					2015				
Variable	Component				Variable	Component			
	C1	C2	C3	C4		C1	C2	C3	C4
T	0.224	−0.371	0.585	−0.517	T	−0.472	0.181	0.022	0.053
pH	0.002	0.099	0.678	0.212	pH	−0.345	−0.143	−0.097	−0.111
Eh	−0.132	0.122	0.852	−0.023	TDS	0.759	0.619	0.131	−0.155
CE	0.865	0.070	−0.064	0.131	Na^+^	0.154	0.900	0.398	0.028
TDS	0.769	0.565	−0.027	0.215	K^+^	0.330	0.469	0.283	0.123
Har	0.091	0.976	0.085	0.105	Ca^2+^	0.877	0.365	0.122	0.159
HCO_3_^−^	0.907	0.041	0.010	−0.042	Mg^2+^	0.884	0.189	0.018	−0.126
Na^+^	0.956	0.030	−0.010	0.114	Li	0.015	0.377	0.601	0.009
K^+^	0.344	0.105	0.215	0.841	HCO_3_^−^	0.081	0.123	0.989	0.022
Ca^2+^	−0.006	0.857	0.165	0.244	Cl^−^	0.845	0.459	0.085	0.253
Mg^2+^	0.191	0.916	−0.031	−0.084					

**Table 4:** Clustering of flows as a result of the cluster analysis for the year, 2005, (Eh = Redox potential, TDS = Total dissolved solids, and Har = Hardness).

Flow	Statistics Data	T °C	pH	Eh mV	EC μs/cm	TDS mg/L	Har mg/L	HCO_3_^−^ mg/L	Na^+^ mg/L	K^+^ mg/L	Ca^2+^ mg/L	Mg^2+^ mg/L	As µg/L	F mg/L
Regional	Mean	25.97	7.71	267.1	503.8	271.9	155.75	285.47	74.67	10.14	47.33	9.09	19.88	0.91
	N	30	30	30	30	30	30	30	30	30	30	30	30	30
	Desv.	1.95	0.537	95.29	347.94	197.49	46.67	225.84	106.8	3.58	14.65	9.8	12.2	0.54
	Mín.	22.4	6.36	14	0.01	152	27	121.51	24	2.7	10.3	0.30	4.6	0.10
	Máx.	30	8.55	392	2150	1260	249.1	1439.21	612.5	22	85	34	61.4	2.6
Local	Mean	18.3	8.05	137.8	553.8	473.8	383	199.4	76.24	13.54	85.56	41.12	8.68	0.42
	N	5	5	5	5	5	5	5	5	5	5	5	5	5
	Desv.	1.04	0.203	187.75	608.59	469.62	447.96	119.9	83	10.5	102.2	47.5	13.52	0.58
	Mín.	17.4	7.8	67	110	61	28.5	36.45	9.2	4.7	9.6	1.1	0.49	0.00
	Máx.	19.7	8.28	341	1561	1110	11587	374.59	197	25	262.8	122	32.7	1.1
Intermediate	Mean	25.02	7.84	275.63	496.97	249.15	188.36	257.55	45.94	10.5	50.53	13.74	15.36	0.62
	N	44	44	44	44	44	44	44	44	44	44	44	44	44
	Desv.	2.14	0.439	100.16	143.24	64.63	156.77	74.85	57.46	26.35	4.6	13.22	10.33	0.42
	Min.	16.2	6.09	10.0	270	168	56.8	72.9	9	2.7	15.5	1.00	4.94	0.03
	Max.	30	8.4	415.00	893	427	417.10	473.16	145	27.7	92.6	54	61.4	1.82
Mixture	Mean	23.81	7.18	188.55	458.7	241.15	158.84	251.08	44.25	9.99	46.06	10.61	18.57	1.03
	N	20	20	20	20	20	20	20	20	20	20	20	20	20
	Desv.	1.95	0.476	121.54	111.07	55.6	58.34	58.18	17.33	2.16	18.08	7.12	8.31	0.353
	Min.	21.1	6.48	45	258	139	89.1	197.15	14.60	5.7	28.2	2.5	4.7	0.10
	Max.	29.2	8.13	376	687	391	369.7	473.16	98	13.2	117.6	25.2	44.5	1.86

**Table 5:** Clustering of flows as a result of the cluster analysis for the year, 2015 (TDS = Total dissolved solids).

Flow	Statistics Data	T °C	pH	TDS mg/L	Na^+^ mg/L	K^+^ mg/L	Ca^2+^ mg/L	Mg^2+^ mg/L	Li mg/L	HCO_3_^−^ mg/L	Cl^−^ mg/L	As µg/L	F mg/L
Regional	Mean	27.68	7.53	221.58	46.06	9.66	29.07	10.23	0.064	215.81	16.54	20.51	1.33
	N	41	41	41	41	41	41	41	41	41	41	59	59
	Desv.	3.22	0.505	47.94	22.82	3.47	11.02	7.99	0.092	52.55	5.406	19.55	0.921
	Min.	22.5	6.71	137.20	17.64	1.06	1.85	0.10	0.02	134.69	8.44	3.64	0.44
	Max.	37.0	8.89	347.90	106.73	17.62	63.97	29.27	0.60	363.07	35.73	121.9	5.40
Local	Mean	23.88	6.66	882.77	152.12	18.8	164.96	56.37	0.178	292.8	250.63	16.94	1.29
	N	7	7	7	7	7	7	7	7	7	7	7	7
	Desv.	2.44	0.284	514.7	71.6	7.22	110.6	69.1	0.159	235.48	181.29	8.99	0.30
	Min.	20.4	6.3	245.49	91.3	7.49	33.67	4.78	0.05	140.3	37.22	7.34	0.80
	Max.	27.8	7.19	161.1	294.03	30.92	374.37	197.5	0.52	816.18	620.3	31.91	1.79
Intermediate	Media	26.04	7.32	253.46	47.58	10.20	40.93	16.26	0.083	227.08	36.39	21.7	1.04
	N	48	48	48	48	48	48	48	48	48	48	48	48
	Desv.	3.36	0.49	121.09	35.18	4.61	29.48	11.97	0.122	104.52	55.42	20.99	0.609
	Min.	19.10	6.43	58.8	12.41	2.03	5.78	1.15	0.01	133.71	8.44	6.25	0.39
	Max.	40.1	8.19	721.77	181.3	23.18	198.92	50.18	0.71	816.18	268.0	241.3	4.25
Mixture	Media	25.99	7.68	217.43	39.58	8.93	29.23	13.39	0.060	213.76	14.91	25.3	1.02
	N	20	20	20	20	20	20	20	20	20	20	20	20
	Desv.	2.21	0.375	72.02	33.12	2.95	9.77	11.12	0.073	66.74	5.66	51.21	0.677
	Min.	21.7	6.59	98.0	18.13	3.99	9.41	0.83	0.01	132.74	8.93	5.19	0.39
	Max.	30.20	8.34	392.0	166.13	13.38	47.24	40.76	0.36	420.9	31.76	120.65	3.60

**Table 6:** Calculation of the Natural background levels (NBLs).

Flows	As µg/L	
Ref [10]	2005	2015
Regional flow	28.4	29.42
Intermediate flow	23.1	31.25
Local flow	4.92	18.28
Mixture	24.5	25.21

**Table 7:** Best-fitted variogram models and their parameters for the indicator values (threshold = REF of As) in the three case studies (C1 = sill; A = range in m; and Nugget = 0).

Year	Flow	Level	Covariance	Model	C1	A	Average Estandar Error	Nugget
2005	Regional	REF	0.1642	Spherical	0.1642	6763.01	0.349	0
		NBL	0.0635	Spherical	0.0635	30,790.62	0.359	0.032
	Intermediate	REF	0.2008	Spherical	0.2008	4929.51	0.399	0
		NBL	0.097	Spherical	0.097	14,903.06	0.291	0.063
	Mixture	REF	0.282	Spherical	0.282	33,408.57	0.277	0.0193
		NBL	0.0798	Spherical	0.0798	5718.988	0.231	0
2015	Regional	REF	0.171	Spherical	0.171	7556.05	0.385	0.0803
		NBL	0.159	Spherical	0.159	3273.94	0.385	0
	Intermediate	REF	0.181	Spherical	0.181	6976.92	0.392	0.042
		NBL	0.111	Spherical	0.111	5718.98	0.319	0.033
	Mixture	REF	0.209	Spherical	0.209	9660.04	0.457	0.133
		NBL	0.136	Spherical	0.136	43,928.76	0.375	0.131

## Supplementary Materials

The following are available online at http://www.mdpi.com/1660-4601/15/11/2374/s1, Table S1: Databases_2005_2015.
